# Patient- and Caregiver-Related Factors Associated with Caregiver Assessed Global Deterioration Scale Scoring in Demented Patients

**DOI:** 10.1155/2018/9396160

**Published:** 2018-06-04

**Authors:** Antonios A. Mougias, Foteini Christidi, Evaggelia Kontogianni, Elena Skaltsounaki, Anastasios Politis, Antonios Politis

**Affiliations:** ^1^Alzheimer Center, Greek Psychogeriatric Association “Nestor”, Athens, Greece; ^2^Medical School, National and Kapodistrian University of Athens, Athens, Greece; ^3^Faculty of Medicine, Comenius University, Bratislava, Slovakia; ^4^1^st^ Department of Psychiatry, Aeiginition Hospital, Medical School, National and Kapodistrian University of Athens, Athens, Greece

## Abstract

**Background:**

Informant-based rating scales are widely used in dementia but patients' and caregivers' features influence the final scoring. We aimed to evaluate the role of patient- and caregiver-related factors in a caregiver rated Global Deterioration Scale (GDS) score in a sample of Greek patients with dementia.

**Methods:**

We included 194 patients with dementia and 194 caregivers/family relatives; Mini-Mental State Examination (MMSE); Neuropsychiatric Inventory (NPI); Katz Index of Activities of Daily Living (K-IADL) were administered to (a) patients and Center for Epidemiologic Studies-Depression (CES-D) Scale; Zarit Burden Interview (ZBI) to (b) caregivers. Participants' demographics and patients' and caregivers' characteristics were entered into a 3-block regression analysis.

**Results:**

The final model explained 55% of the total variance of the caregiver assessed GDS score. The following variables significantly contributed to the final model: MMSE (*β*=-0.524); K-IADL (*β=*-0.264); ZBI (*β*=0.145).

**Conclusion:**

We herein confirm the contribution of patients' cognitive and functional status and caregivers' burden in caregiver rated GDS scoring irrespective of demographic-related characteristics.

## 1. Introduction

Global severity measures, including Global Deterioration Scale (GDS) [1], are commonly used in neurodegenerative research to categorize subjects in clinical trials and further evaluate the therapeutic efficacy of both pharmacological and nonpharmacological interventions in relation to the progression of dementia process [2]. It has been suggested that global severity scales are potentially “less influenced by factors such as education, occupation, practice effects, personal background, cultural factors and linguistic factors” that affect mental status and psychometric measures. However, these scales are based on “information about subjective complaints of memory deficit, objective observation of deficit on careful clinical interview, and on assessment of functional ability of the subject” [3] based in part on information from a knowledgeable informant. Herein, we investigated an informant-based version of the GDS, which does not use the direct patient interview procedures conventionally applied for this assessment, for example, in the worldwide approvals of currently used medications for the treatment of Alzheimer's disease (AD) (e.g., rivastigmine and memantine) and in worldwide clinical practice. In general, informant's based rating scales can be influenced by both patients' mental and neuropsychiatric status [4], as well as informant-/caregiver-related factors, including but not limited to depression and burden [5].

In the case of Greece, approximately 200.000 patients have been diagnosed with dementia of the AD type and 50.000 patients still remain undiagnosed [6] while the majority of patients have a low educational level [7]. Apart from patients' neuropsychiatric status and caregivers' mood status and burden, it is still understudied whether patients' and informants' demographic factors including educational level influence GDS score and whether family relationships between patients' and their family caregivers are another influential factor. The latter could be of major importance considering family bonds and structure related to sociocultural values.

The aim of the present study was to evaluate patient- and caregiver-related features in order to identify the predictors that explain the variability of an informant-based GDS score in a large sample of Greek patients with dementia.

## 2. Materials and Methods

### 2.1. Participants

**Figure 1:**
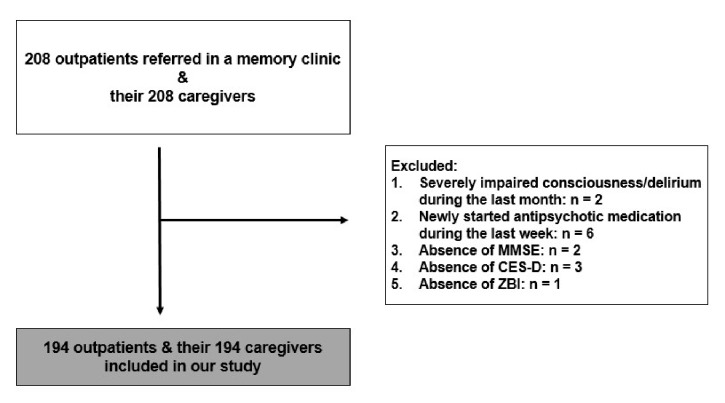
Flow chart with the participants of the present study.

The study included 194 outpatients who had been referred to our memory clinic (“Nestor” Greek Psychogeriatric Association) during the previous 12 months, as well as their 194 responsible caregivers. Inclusion criteria were a diagnosis of AD, or vascular dementia [DSM-IV] [8], or Dementia with Lewy Bodies [9], or frontotemporal dementia [10]. Exclusion criteria were as follows: permanent or transient severely impaired consciousness or delirium during the previous month; newly started antipsychotic medication during the last week before test administration; absence of available data in psychometric scales (Figure 1). All patients if possible and their caregivers were fully informed about the purpose of the study and provided written informed consent. The study was approved by the Local Ethical Committee of the Psychogeriatric Association and was conducted in accordance with the Declaration of Helsinki, as revised in 1983.

### 2.2. Testing Materials

The Mini-Mental State Examination (MMSE) was administered to patients [11, 12] and had a MMSE score ≤ 24. Dementia staging was defined based on the GDS [1] (translated and validated in Greek after permission by authors of the original scale) that assesses dementia severity over seven levels, i.e., GDS 1: absence of cognitive changes to GDS 7: very severe cognitive deficits. The score was derived from the caregiver interview [1]. Patients' neuropsychiatric symptoms and activities of daily living were evaluated using the Neuropsychiatric Inventory (NPI) [13, 14] and the Katz Index of Independence in Activities of Daily Living (K-IADL) [15], respectively, completed by their caregivers. All caregivers were given the following self-administered scales: (a) the Center for Epidemiological Studies-Depression (CES-D) Scale [16, 17] for the evaluation of depressive symptoms; (b) the Zarit Burden Inventory (ZBI) [18, 19] for the assessment of the subjective burden experienced by dementia patients' caregivers. The following demographic characteristics for all patients were also included for the purposes of the present study: age, gender, years of education, and dementia type. Furthermore, the following characteristics were recorded with regard to caregiver/caregiving: age, gender, years of education, weekly caregiving time, caregivers being a spouse, and caregiver living with the patient.

### 2.3. Statistical Analysis

Psychometric variables of interest were examined for normality (skewness, Q-Q plots, and Kolmogorov-Smirnov test) and assumptions for normality were not violated. A 3-block hierarchical regression analysis was conducted to identify the contribution of patients' and caregivers characteristics to GDS scoring. Multicollinearity was checked for regression analysis and assumptions (tolerance; VIF) were not violated. Patients' and caregivers' demographic features (age, gender, years of education, and caregiver being a spouse) were entered in block 1, followed by patients' disease and clinical/psychometric characteristics (diagnosis of AD, MMSE, NPI, and K-IADL) in block 2, and caregiving characteristics (CES-D, ZBI, weekly hours of caregiving, presence of professional caregiver, and caregiver living with the patient) in block 3. All statistical analyses were conducted using IBM SPSS v. 22.0 and the level of significance was set at* p*<0.05.

## 3. Results

### 3.1. Descriptive Characteristics of the Patients' and Caregivers' Samples

Table 1 presents demographic, disease-related, and psychometric characteristics for the sample. The total sample of patients represented a typical community dementia sample visiting an outpatient clinic offering services towards dementia patients' need. Patients were elderly, mainly female with a relatively low level of education. Most patients were diagnosed with AD (N = 131; 67.53%) whereas non-AD diagnosis in the present sample included vascular dementia (N = 24; 12.37%) or not otherwise specified dementia (N = 39; 20.10%). Most patients had moderate to severe dementia, according to the MMSE score (MMSE 0-9, N = 76, 39.2%; MMSE 10-19, N = 97, 50.0%; MMSE 20-23, N = 21, 10.8%). Caregivers were younger and more educated than the patients (p < 0.001); most of them were living in the same house or building with the patients and were involved in caregiving on a daily basis. Professional caregiving as a support to the family caregiving was also reported in 54 / 194 patients (27.8%). General cognitive status was impaired (mean MMSE = 11.07); only 2 patients did not have any behavioral problem on the NPI. Furthermore, 60 caregivers (30.9%) had depression based on the CES-D (cut-off ≥24) while more than half (N = 99, 51.0%) were highly burdened based on ZBI (cut-off >44).

### 3.2. Contribution of Patients' and Caregivers' Characteristics to GDS Scoring: Regression Analysis

**Figure 2:**
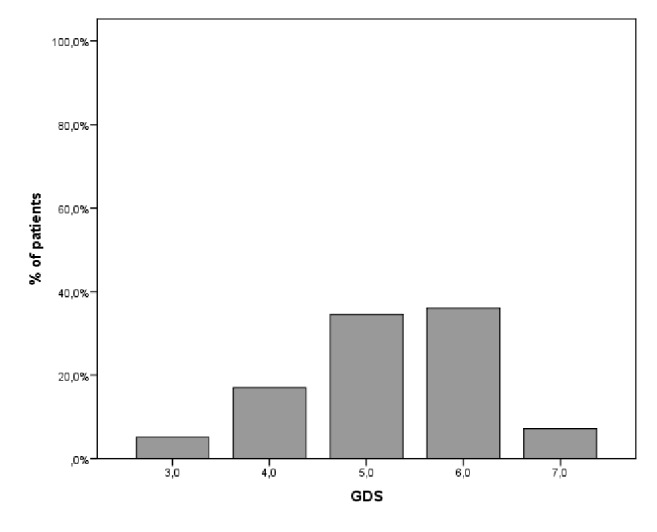
Distribution of GDS in the total sample of dementia patients based on their caregivers' scoring.

Figure 2 shows the distribution of GDS based on caregivers scoring. The following frequencies were observed for each scoring stage: GDS 3: N = 10 (5.2%); GDS 4: N = 33 (17.0%); GDS 5: N = 67 (34.5%); GDS 6: N = 70 (36.1%); GDS 7: N = 14 (7.2%).

Table 2 summarizes regression analysis findings regarding the contribution of patient- and caregiver-related characteristics on the GDS scoring. Demographics (block 1) did not significantly contribute to GDS scoring (adjusted *R*^2^ = 0.005;* F* = 1.133;* p* = 0.344,* ns*). The inclusion of patient-related disease and clinical/psychometric characteristics resulted in a significant increase in R^2^ (Δ*R* = 0.520;* ΔF* = 53.920;* p* < 0.001), with the model significantly explaining 53.5% of the GDS variance (adjusted* R*^*2*^ = 0.535;* F* = 21.149;* p* < 0.001). MMSE (*β* = -0.528;* p* < 0.001) and K-IADL (*β* = -0.310;* p* < 0.001) significantly contributed to the model. Finally (Model 3), the inclusion of caregiving features resulted in a marginal increase in the *R*^2^ (*ΔR* = 0.026;* ΔF* = 2.249;* p* = 0.052), with the final model being significant (*F* = 15.742;* p* < 0.001) and explaining 55.0% of the GDS variance (adjusted *R*^2^ = 0.550). The following variables emerged as significant predictors in the final model: MMSE (*β* = -0.524;* p* < 0.001), K-IADL (*β* = -0.264;* p* < 0.001), and ZBI (*β* = 0.145;* p* < 0.05).

## 4. Discussion

In this study, we investigated the contribution of demographic factors, patients' cognitive and behavioral characteristics, and caregivers' depression and burden status, as well as general caregiving features related to family relationships in the scoring of a global assessment scale such as the GDS. To the best of our knowledge, this is the first study that addresses the issue of caregiver scoring of the GDS as well as associated factors. We found that patients' MMSE and K-IADL and caregivers' ZBI scores were significant factors in the final model when demographics and patients' and caregivers' features were all included as prior variables which were accounted for.

The GDS is one of the most commonly used global assessment scales for measuring patients' severity. It has been found to be advantageous to overall subject assessment in terms of utility in incipient and severe AD, reliability, sensitivity to AD course [2], and response to cognitive enhancers (e.g., rivastigmine [20]). Furthermore, as with other global assessment scales, it has been considered to be less influenced by demographic, cultural, and linguistic factors, patients' occupation, and personal background and practice effects [2]. These properties constitute a clear advantage when examining patients with low educational background, as it is the case in Greek patients with dementia [7]. In the present study, neither patients' nor caregivers' demographic characteristics (i.e., age, gender, and years of education) significantly explained the variance of GDS scoring, in accordance with previous studies using other informant-based rating scales for patients' functional abilities [21]. Others have found that caregivers' higher educational and sociocultural level is associated with more accurate reports of patients' functioning [22] or that gender may influence functional assessment of patients' basic and instrumental activities of daily living, behavioral and psychological symptoms, anosognosia, and quality of life [23].

Patients' clinical and psychometric data explained 53.5% of the variance on the GDS score in our study, with MMSE and K-IADL significantly contributing to the model. Higher MMSE and K-IADL scores were predictive of lower GDS scores. The significant contributions of MMSE and K-IADL are in accordance with other studies in patients with mild deficits; caregivers often overestimate functional abilities of patients whose cognitive impairment is mild [24]. Negative findings have also been detected [25].

Caregivers' information should always be complementary to patients' medical history and clinical/psychometric examination [26, 27]. Thus, factors that could potentially influence caregivers' rating of patients' severity and functional status cannot be ignored in clinical practice or in research. In the present study, we found that caregivers'/caregiving features marginally increase the percent of GDS variance in the final regression analysis (Model 3) with the final model explaining 55% of the total variance. Caregiver burden [23, 28, 29], depression [23, 30], and poor quality of life [31, 32] have been often considered as confounders in the estimation of patients' clinical profile, including neuropsychiatric symptoms, cognitive severity, daily living functional activities, and quality of life. Even though we found a significant contribution of the caregivers' burden, we failed to identify a significant contribution with respect to caregiver depression in contrast with previous studies [28, 33] but in accordance with others which did not report significant effects [21]. In contrast to other studies that report a relationship between the patient and the caregiver and the potential bias in rating of informant-based scales for patients [34, 35], our findings did not support previous studies. None of the related factors (caregiver being as spouse, caregiver living with the patient) significantly contributed to regression models.

Limitations of the present study are the inclusion of a sample of patients/caregivers from a specialized outpatient memory clinic which might result in bias regarding higher awareness of cognitive and functional decline, as well as the inclusion of consecutive patients with the majority having a diagnosis of AD which did not permit the evaluation of the contribution of patients' and caregivers' characteristics to GDS scoring in other dementias with pronounced behavioral symptoms (e.g., frontotemporal dementia). We also acknowledge some methodological issues regarding the evaluation of caregivers' characteristics, including hours of caregiving. Hours of daily care may considerably vary within a week and this variability (which was out of the main aim of the present study) may affect caregiver burden and depression. Furthermore, we assume that several other factors that were not included in the current study might further contribute to the GDS score. For instance, we did not include psychometric questionnaires to assess caregivers' temperament and character features that might further contribute to their attitude towards patients' cognitive and functional status and their judgement regarding severity of patient's deterioration and the final GDS scoring. However, patients and caregivers characteristics as the ones examined in the current analysis are some of the most commonly recorded data in outpatient geriatric clinics. The fact that 55% of the GDS variance is explained by these factors and that caregivers' burden significantly contributes to the final model apart from patients' cognitive and functional status has significant clinical implications when considering GDS score based on caregivers' interview. Further endeavors to expand our understanding of the different factors contributing to informants' rating in different global assessment scales and how these factors influence or are associated with different outcomes at the time of diagnosis and after pharmacological and nonpharmacological interventions are warranted. Additionally, considering that in the past few years the enduring recession of Greece has been found to impinge on the mental health of the general population [36] and that one of the critical concerns of dementia caregivers is the financial strain they experience [37], future directions should include socioeconomic data regarding both patients' and their caregivers'.

## 5. Conclusions

In conclusion, we herein confirm the contribution of patients' cognitive and functional status and caregivers' burden to caregiver rated GDS scoring independent of demographic-related characteristics. Considering that increased caregivers' psychological burden has been associated with a faster time to institutionalization and death in dementia patients [38, 39], clinicians and researchers should consider caregivers' burden when interpreting dementia severity scales scored by caregivers.

## Figures and Tables

**Table 1:** Demographic, clinical, and psychometric characteristics for the samples of patients with caregivers.

**Variables**	**Patients**		**Caregivers**	
	**Mean ± SD**	**Min-Max**	**Mean ± SD**	**Min-Max**
Age (yrs)	79.06 ± 7.16	56-97	59.40 ± 12.96	24-87
Gender (M / F)	65 / 129	-	60 / 134	-
Education (yrs)	7.57 ± 4.02	0-16	11.58 ± 3.82	2-20
Dementia type (AD / non-AD)	131 / 63	-	-	-
MMSE	11.07 ± 6.64	0-23	-	-
NPI	33.06 ± 19.02	0-88	-	-
K-IADL	3.50 ± 2.00	0-6	-	-
GDS	5.23 ± 0.99	3-7	-	-
CES-D	-	-	19.64 ± 10.05	1-51
ZBI	-	-	43.42 ± 46.42	8-74
Weekly caregiving (hrs)	-	-	101.08 ± 69.70	0-168
Professional caregiver (Y / N)	-	-	54 / 140	-
Caregiver being a spouse (Y / N)	-	-	77 / 117	-
Caregiver living with the patient (Y / N)	-	-	124 / 70	-

*Note. *AD = Alzheimer's dementia; MMSE = Mini-Mental State Examination; NPI = Neuropsychiatric Inventory; K-IADL = Katz Index of Activities of Daily Living; GDS = Global Deterioration Scale; CES-D = Center for Epidemiological Studies-Depression; ZBI = Zarit Burden Inventory; M / F = male / female; Y / N = yes / no; Yrs = years; hrs = hours.

**Table 2:** Summary of hierarchical regression analysis to identify the contribution of patients' and caregivers' characteristics on GDS scoring.

**Variables**	**Model 1**	**Model 2**	**Model 3**
	**β**	**β**	**β**
**Demographic characteristics**			
Patient's Age	-0.084	-0.005	-0.007
Patient's Gender	0.138	0.115	0.120
Patient's Education	0.007	0.019	0.027
Caregiver's Age	-0.115	-0.043	-0.033
Caregiver's Gender	0.086	0.080	0.078
Caregiver's Education	-0.104	-0.076	-0.086
Caregiver being a spouse	-0.125	-0.078	-0.056
**Patients' disease and clinical/psychometric features**			
Diagnosis of AD		-0.055	-0.064
MMSE		-0.528*∗∗∗*	-0.524*∗∗∗*
NPI		-0.041	-0.108
K-IADL		-0.310*∗∗∗*	-0.264*∗∗∗*
**Caregivers' psychometric features and caregiving characteristics**			
CES-D			0.013
ZBI			0.145*∗*
Weekly hours of caring			-0.059
Caregiver living with the patient			0.083
Presence of professional caregiver			-0.108

***F*-model**	1.133	21.149*∗∗∗*	15.742*∗∗∗*
***R*** ^***2***^	0.041	0.561	0.587
**Adjusted *R*** ^***2***^	0.005	0.535	0.550
Δ***R***^***2***^	0.041	0.520	0.026
Δ***F***	1.133	53.920*∗∗∗*	2.249

*Note*. AD = Alzheimer's dementia; MMSE = Mini-Mental State Examination; NPI = Neuropsychiatric Inventory; K-IADL = Katz Index of Activities of Daily Living; GDS = Global Deterioration Scale; CES-D = Center for Epidemiological Studies-Depression; ZBI = Zarit Burden Inventory. *β* corresponds to standardized beta coefficients. Bold adjusted *R*^2^ correspond to models significantly explaining the variance of the dependent variable (i.e., GDS scoring). *∗p* < 0.05; *∗∗p* < 0.01; *∗∗∗p* < 0.001.

## Data Availability

The data used to support the findings of this study are available from the corresponding author upon request.

## References

[1] Reisberg B., Ferris S. H., De Leon M. J., Crook T. (1982). The global deterioration scale for assessment of primary degenerative dementia. *The American Journal of Psychiatry*.

[2] Reisberg B. (2007). Global measures: Utility in defining and measuring treatment response in dementia. *International Psychogeriatrics*.

[3] Reisberg B., Ferris S. H., de Leon M. J. (1988). Stage‐specific behavioral, cognitive, and in vivo changes in community residing subjects with age‐associated memory impairment and primary degenerative dementia of the Alzheimer type. *Drug Development Research*.

[4] Martyr A., Nelis S. M., Clare L. (2014). Predictors of perceived functional ability in early-stage dementia: self-ratings, informant ratings and discrepancy scores. *International Journal of Geriatric Psychiatry*.

[5] Argüelles S., Loewenstein D. A., Eisdorfer C., Argiielles T. (2001). Caregivers' judgments of the functional abilities of the Alzheimer's disease patient: Impact of caregivers' depression and perceived burden. *Journal of Geriatric Psychiatry and Neurology*.

[6] Alzheimer Europe The prevalence of dementia in Europe: Greece. http://www.alzheimer-europe.org/Policy-in-Practice2/Country-comparisons/2013-The-prevalence-of-dementia-in-Europe/Greece.

[7] Jelastopulu E., Giourou E., Argyropoulos K. (2014). Demographic and Clinical Characteristics of Patients with Dementia in Greece. *Advances in Psychiatry*.

[8] American Psychiatric Association (2000). *Diagnostic and Statistical Manual of Mental Disorders*.

[9] McKeith I. G., Galasko D., Kosaka K. (1996). Consensus guidelines for the clinical and pathologic diagnosis of dementia with Lewy bodies (DLB): report of the consortium on DLB international workshop. *Neurology*.

[10] Neary D., Snowden J. S., Gustafson L. (1998). Frontotemporal lobar degeneration: a consensus on clinical diagnostic criteria. *Neurology*.

[11] Folstein M. F., Folstein S. E., McHugh P. R. (1975). “Mini mental state”. A practical method for grading the cognitive state of patients for the clinician. *Journal of Psychiatric Research*.

[12] Fountoulakis K. N., Tsolaki M., Chantzi H., Kazis A. (2000). Mini mental state examination (MMSE): a validation study in Greece. *American Journal of Alzheimer’s Disease & Other Dementias*.

[13] Cummings J. L., Mega M., Gray K., Rosenberg-Thompson S., Carusi D. A., Gornbein J. (1994). The neuropsychiatric inventory: comprehensive assessment of psychopathology in dementia. *Neurology*.

[14] Politis A. M., Mayer L. S., Passa M., Maillis A., Lyketsos C. G. (2004). Validity and reliablity of the newly translated Hellenic Neuropsychiatric Inventory (H-NPI) applied to Greek outpatients with Alzheimer's disease: A study of disturbing behaviors among referrals to a memory clinic. *International Journal of Geriatric Psychiatry*.

[15] Katz S., Downs T. D., Cash H. R., Grotz R. C. (1970). Progress in development of the index of ADL. *The Gerontologist*.

[16] Radloff L. S. (1977). The CES-D scale: a self-report depression scale for researching the general population. *Application of Psychological Measures*.

[17] Fountoulakis K., Iacovides A., Kleanthous S. (2001). Reliability, validity and psychometric properties of the Greek translation of the Center for Epidemiological Studies-Depression (CES-D) Scale. *BMC Psychiatry*.

[18] Zarit S. H., Reever K. E., Bach-Peterson J. (1980). Relatives of the impaired elderly: correlates of feelings of burden. *The Gerontologist*.

[19] Mougias A. A., Politis A., Mougias M. A. (2015). The burden of caring for patients with dementia and its predictors. *Psychiatrike = Psychiatriki*.

[20] Corey-Bloom J., Anand R., Veach J. (1998). A randomized trial evaluating the efficacy and safety of ENA 713 (rivastigmine tartrate), a new acetylcholinesterase inhibitor, in patients with mild to moderately severe Alzheimer's disease. *International Journal of Geriatric Psychopharmacology*.

[21] Loewenstein D. A., Arguelles S., Bravo M. (2001). Caregivers' Judgments of the Functional Abilities of the Alzheimer's Disease Patient: A Comparison of Proxy Reports and Objective Measures. *The Journals of Gerontology Series B: Psychological Sciences and Social Sciences*.

[22] Dassel K. B., Schmitt F. A. (2008). The impact of caregiver executive skills on reports of patient functioning. *The Gerontologist*.

[23] Conde-Sala J. L., Reñé-Ramírez R., Turró-Garriga O. (2013). Factors associated with the variability in caregiver assessments of the capacities of patients with alzheimer disease. *Journal of Geriatric Psychiatry and Neurology*.

[24] Doble S. E., Fisk J. D., Rockwood K. (1999). Dementia: Assessing the ADL functioning of persons with Alzheimer's disease: Comparison of family informants' ratings and performance-based assessment findings. *International Psychogeriatrics*.

[25] Karagiozis H., Gray S., Sacco J., Shapiro M., Kawas C. (1998). The Direct Assessment of Functional Abilities (DAFA): A comparison to an indirect measure of instrumental activities of daily living. *The Gerontologist*.

[26] McKhann G. M., Knopman D. S., Chertkow H. (2011). The diagnosis of dementia due to Alzheimer's disease: Recommendations from the National Institute on Aging-Alzheimer's Association workgroups on diagnostic guidelines for Alzheimer's disease. *Alzheimer’s & Dementia*.

[27] Waldemar G., Dubois B., Emre M. (2007). Recommendations for the diagnosis and management of Alzheimer's disease and other disorders associated with dementia: EFNS guideline. *European Journal of Neurology*.

[28] Zanetti O., Geroldi C., Frisoni G. B., Bianchetti A., Trabucchi M. (1999). Contrasting results between caregiver's report and direct assessment of activities of daily living in patients affected by mild and very mild dementia: The contribution of the caregiver's personal characteristics. *Journal of the American Geriatrics Society*.

[29] Mangone C., Sanguinetti R., Baumann P. (1993). Influence of Feelings of Burden on the Caregiver's Perception of the Patient's Functional Status. *Dementia and Geriatric Cognitive Disorders*.

[30] Pfeifer L., Drobetz R., Fankhauser S., Mortby M. E., Maercker A., Forstmeier S. (2013). Caregiver rating bias in mild cognitive impairment and mild Alzheimer's disease: Impact of caregiver burden and depression on dyadic rating discrepancy across domains. *International Psychogeriatrics*.

[31] Schulz R., Cook T. B., Beach S. R. (2013). Magnitude and causes of bias among family caregivers rating Alzheimer disease patients. *The American Journal of Geriatric Psychiatry*.

[32] Arons A. M., Krabbe P. F., Schölzel-Dorenbos C. J., Van Der Wilt G. J., Rikkert M. G. O. (2013). Quality of life in dementia: A study on proxy bias. *BMC Medical Research Methodology*.

[33] Shega J. W., Hougham G. W., Stocking C. B., Cox-Hayley D., Sachs G. A. (2005). Factors associated with self- and caregiver report of pain among community-dwelling persons with dementia. *Journal of Palliative Medicine*.

[34] McLoughlin D. M., Cooney C., Holmes C., Levy R. (1996). Carer informants for dementia sufferers: Carer awareness of cognitive impairment in an elderly community-resident sample. *Age and Ageing*.

[35] Persson K., Brækhus A., Selbæk G., Kirkevold Ø., Engedal K. (2015). Burden of Care and Patient's Neuropsychiatric Symptoms Influence Carer's Evaluation of Cognitive Impairment. *Dementia and Geriatric Cognitive Disorders*.

[36] Economou M., Peppou L. E., Souliotis K., Stylianidis S. (2016). The impact of the economic crisis in Greece: Epidemiological perspective and community implications. *Social and Community Psychiatry: Towards a Critical, Patient-Oriented Approach*.

[37] Dimakopoulou E., Sakka P., Efthymiou A., Karpathiou N., Karydaki M. (2015). Evaluating the needs of dementia patients caregivers in Greece: a questionnaire survey. *Int J Car Sci*.

[38] Brodaty H., Peters K. E., Harris L., Mcgilchrist C. (1993). Time Until Institutionalization and Death in Patients with Dementia: Role of Caregiver Training and Risk Factors. *JAMA Neurology*.

[39] Balardy L., Voisin T., Cantet C., Vellas B. (2005). Predictive factors of emergency hospitalisation in Alzheimer's patients: Results of one-year follow-up in the REAL.FR cohort. *The Journal of Nutrition, Health & Aging*.

